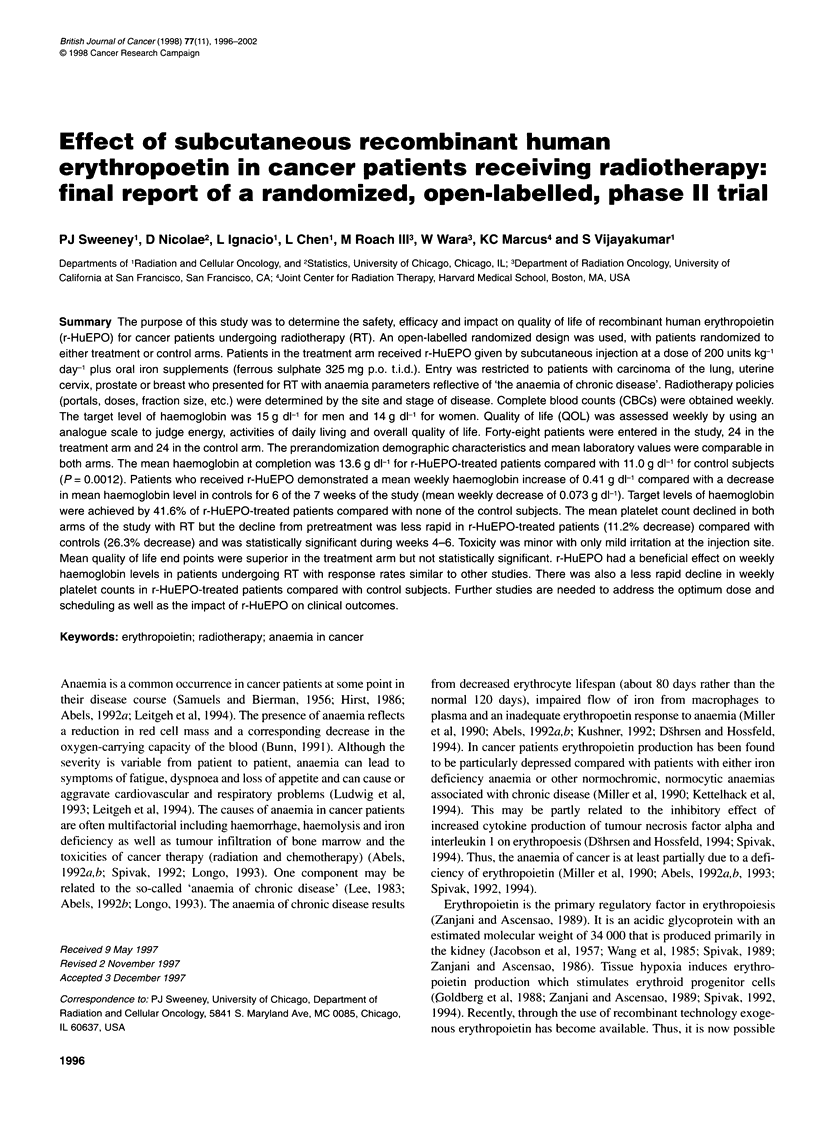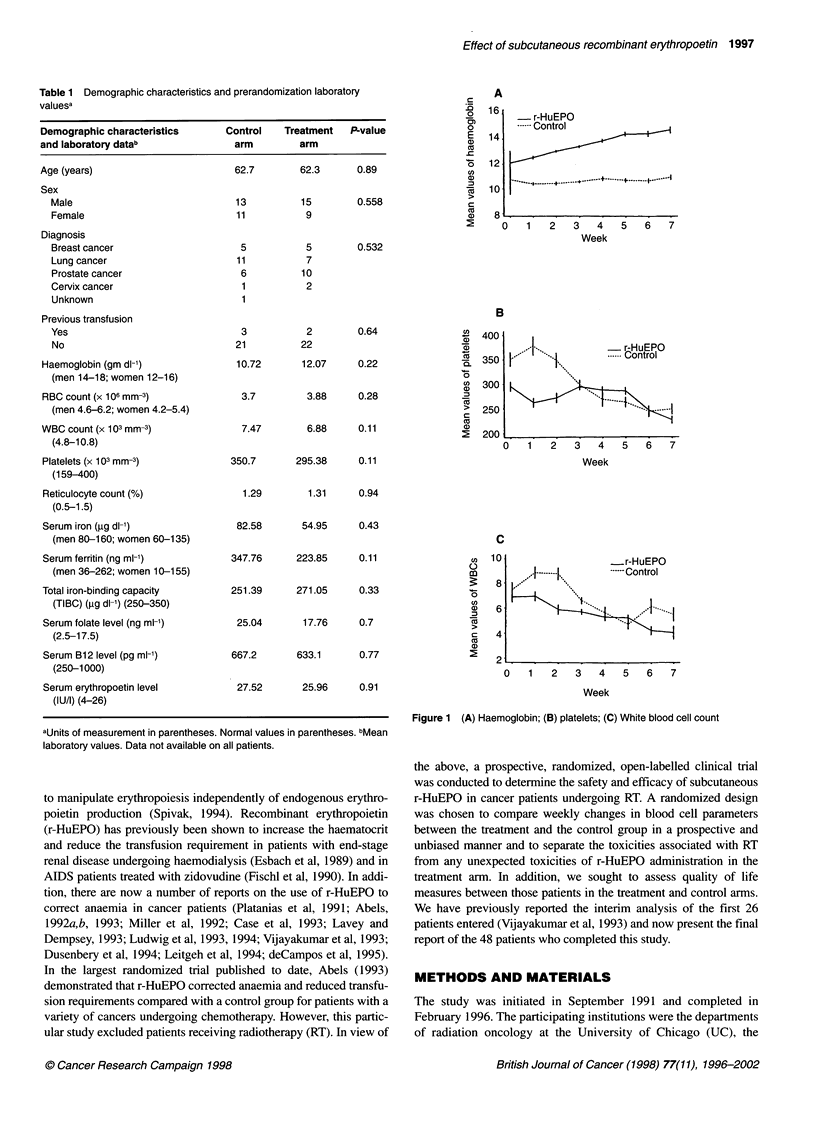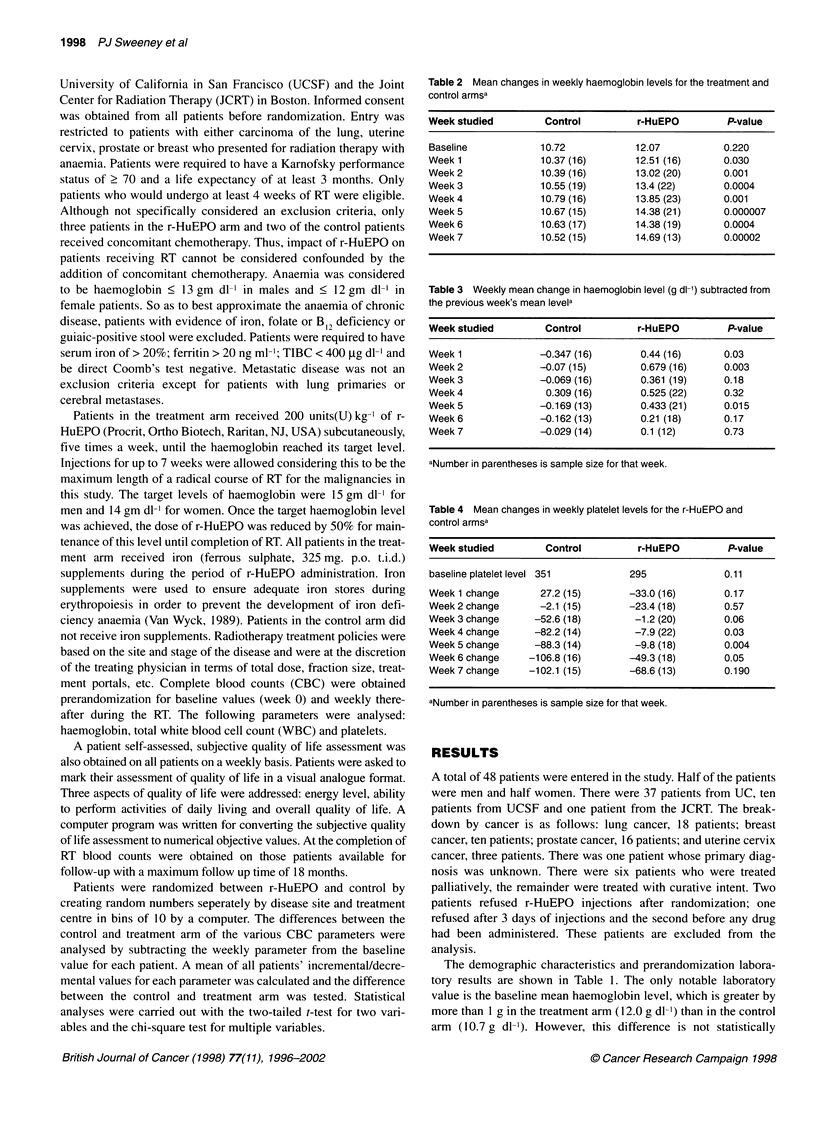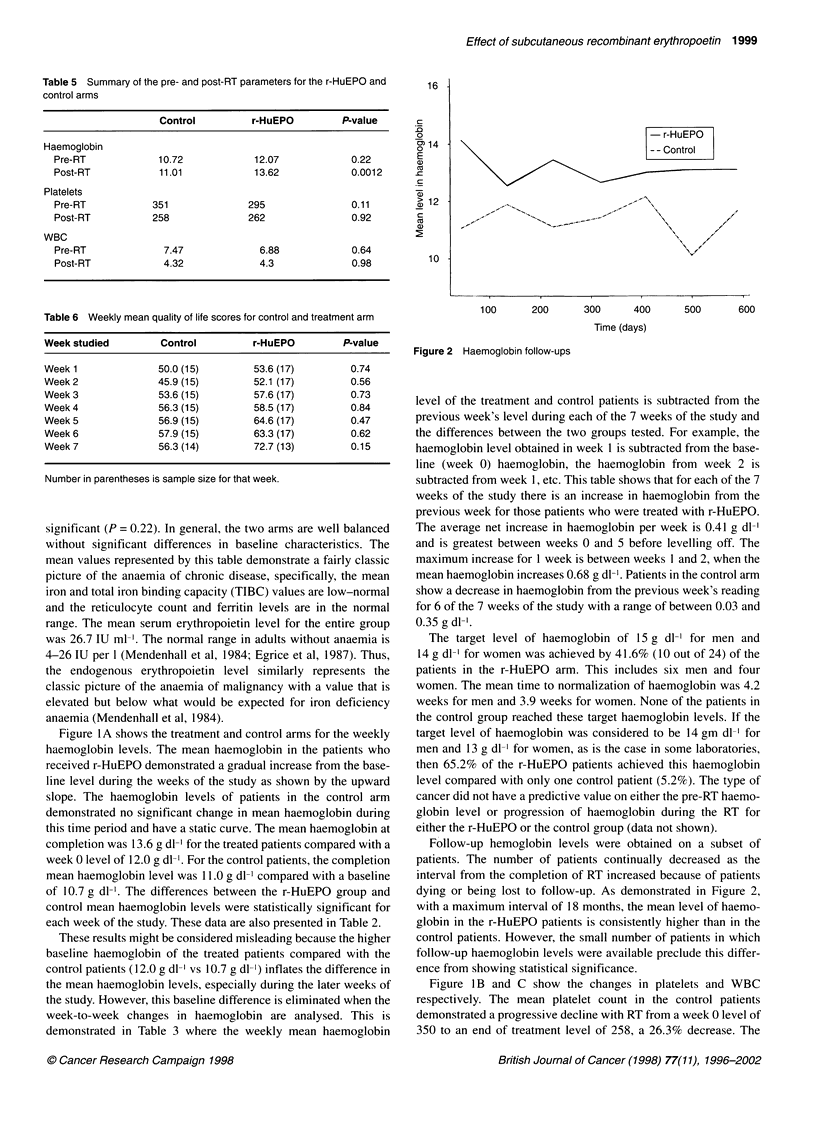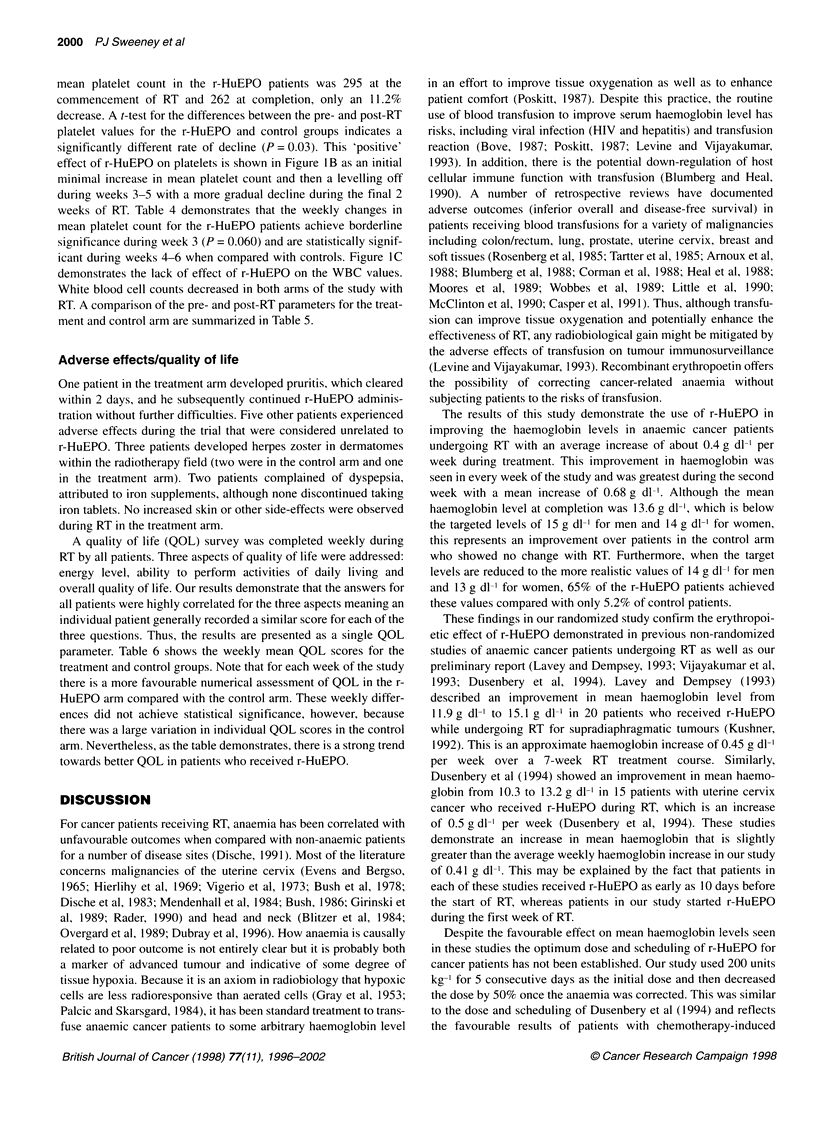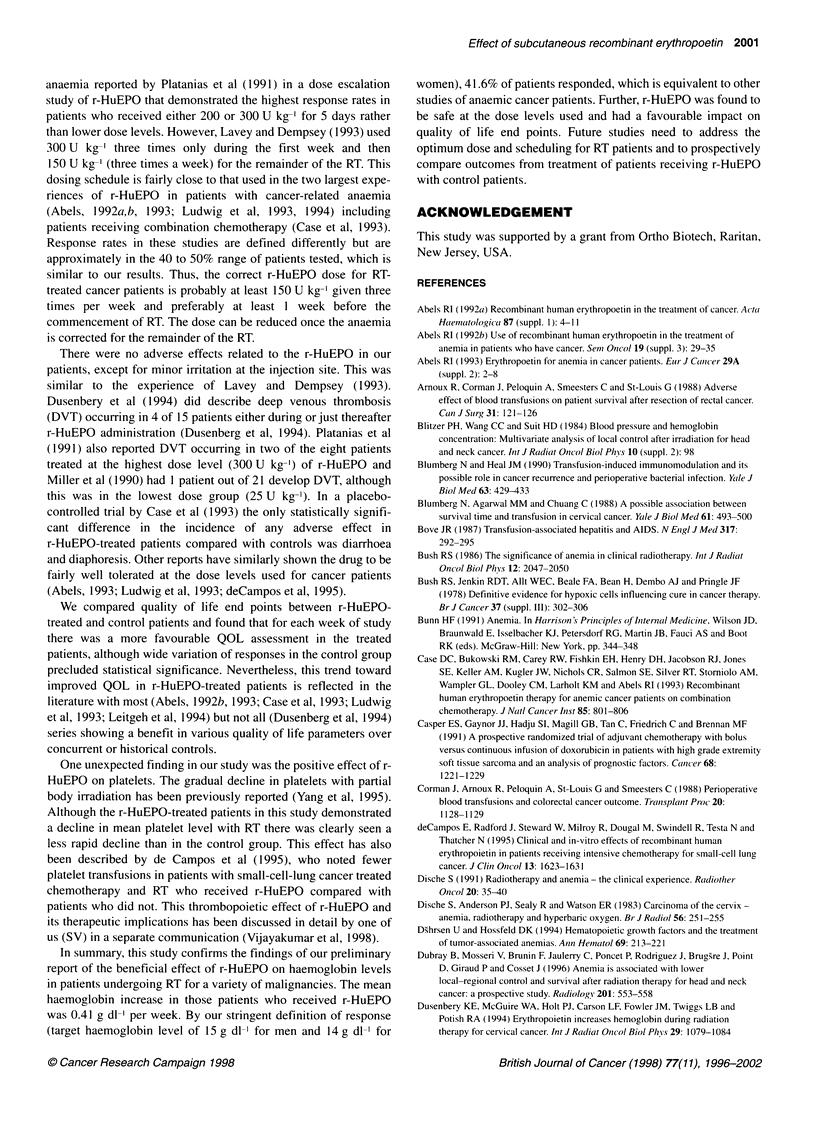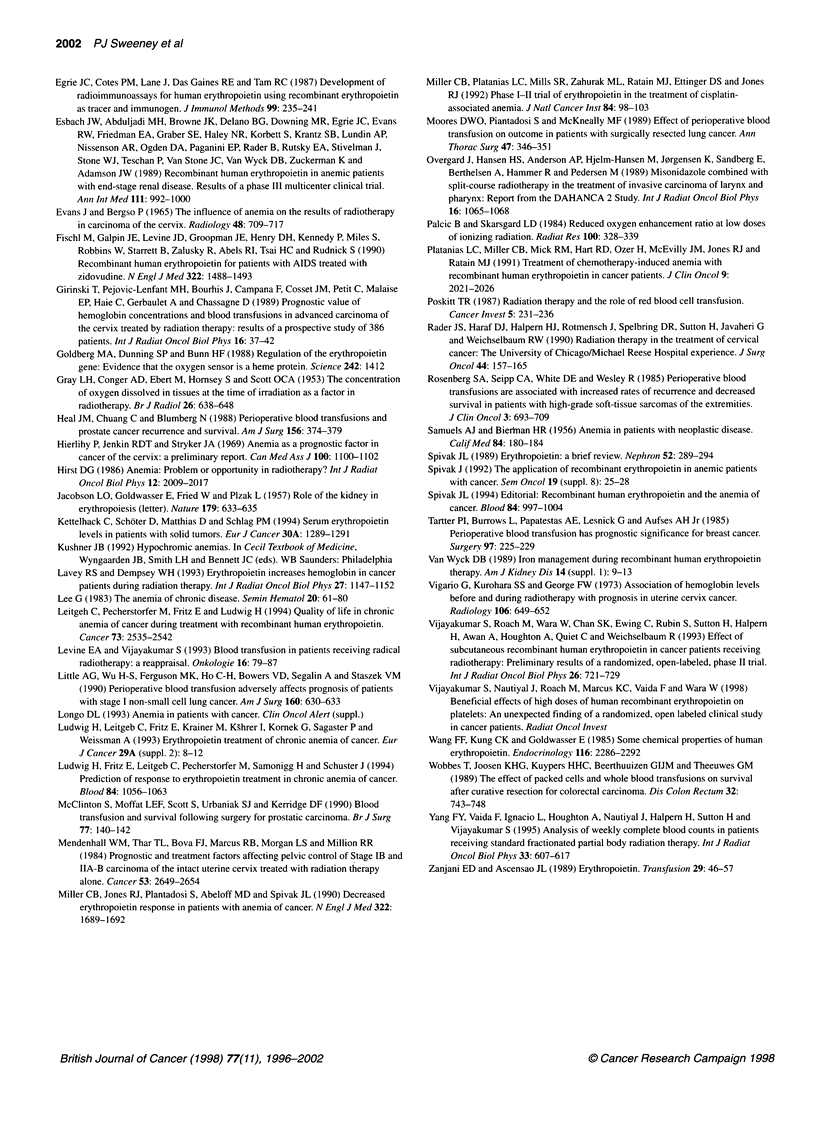# Effect of subcutaneous recombinant human erythropoietin in cancer patients receiving radiotherapy: final report of a randomized, open-labelled, phase II trial.

**DOI:** 10.1038/bjc.1998.331

**Published:** 1998-06

**Authors:** P. J. Sweeney, D. Nicolae, L. Ignacio, L. Chen, M. Roach, W. Wara, K. C. Marcus, S. Vijayakumar

**Affiliations:** Department of Radiation and Cellular Oncology, University of Chicago, IL, USA.

## Abstract

The purpose of this study was to determine the safety, efficacy and impact on quality of life of recombinant human erythropoietin (r-HuEPO) for cancer patients undergoing radiotherapy (RT). An open-labelled randomized design was used, with patients randomized to either treatment or control arms. Patients in the treatment arm received r-HuEPO given by subcutaneous injection at a dose of 200 units kg(-1) day(-1) plus oral iron supplements (ferrous sulphate 325 mg p.o. t.i.d.). Entry was restricted to patients with carcinoma of the lung, uterine cervix, prostate or breast who presented for RT with anaemia parameters reflective of 'the anaemia of chronic disease'. Radiotherapy policies (portals, doses, fraction size, etc.) were determined by the site and stage of disease. Complete blood counts (CBCs) were obtained weekly. The target level of haemoglobin was 15 g dl(-1) for men and 14 g dl(-1) for women. Quality of life (QOL) was assessed weekly by using an analogue scale to judge energy, activities of daily living and overall quality of life. Forty-eight patients were entered in the study, 24 in the treatment arm and 24 in the control arm. The prerandomization demographic characteristics and mean laboratory values were comparable in both arms. The mean haemoglobin at completion was 13.6 g dl(-1) for r-HuEPO-treated patients compared with 11.0 g dl(-1) for control subjects (P = 0.0012). Patients who received r-HuEPO demonstrated a mean weekly haemoglobin increase of 0.41 g dl(-1) compared with a decrease in mean haemoglobin level in controls for 6 of the 7 weeks of the study (mean weekly decrease of 0.073 g dl(-1)). Target levels of haemoglobin were achieved by 41.6% of r-HuEPO-treated patients compared with none of the control subjects. The mean platelet count declined in both arms of the study with RT but the decline from pretreatment was less rapid in r-HuEPO-treated patients (11.2% decrease) compared with controls (26.3% decrease) and was statistically significant during weeks 4-6. Toxicity was minor with only mild irritation at the injection site. Mean quality of life end points were superior in the treatment arm but not statistically significant. r-HuEPO had a beneficial effect on weekly haemoglobin levels in patients undergoing RT with response rates similar to other studies. There was also a less rapid decline in weekly platelet counts in r-HuEPO-treated patients compared with control subjects. Further studies are needed to address the optimum dose and scheduling as well as the impact of r-HuEPO on clinical outcomes.


British Journal of Cancer (1998) 77(11), 1996–2002
© 1998 Cancer Research Campaign

### Effect of subcutaneous recombinant human erythropoetin in cancer patients receiving radiotherapy: final report of a randomized, open-labelled, phase II trial

**PJ Sweeney[1], D Nicolae[2], L Ignacio[1], L Chen[1], M Roach III[3], W Wara[3], KC Marcus[4] and S Vijayakumar[1]**

Departments of [1]Radiation and Cellular Oncology, and [2]Statistics, University of Chicago, Chicago, IL; [3]Department of Radiation Oncology, University of California at San Francisco, San Francisco, CA; [4]Joint Center for Radiation Therapy, Harvard Medical School, Boston, MA, USA

**Summary** The purpose of this study was to determine the safety, efficacy and impact on quality of life of recombinant human erythropoietin (r-HuEPO) for cancer patients undergoing radiotherapy (RT). An open-labelled randomized design was used, with patients randomized to either treatment or control arms. Patients in the treatment arm received r-HuEPO given by subcutaneous injection at a dose of 200 units $kg^{-1}$ $day^{-1}$ plus oral iron supplements (ferrous sulphate 325 mg p.o. t.i.d.). Entry was restricted to patients with carcinoma of the lung, uterine cervix, prostate or breast who presented for RT with anaemia parameters reflective of 'the anaemia of chronic disease'. Radiotherapy policies (portals, doses, fraction size, etc.) were determined by the site and stage of disease. Complete blood counts (CBCs) were obtained weekly. The target level of haemoglobin was 15 g $dl^{-1}$ for men and 14 g $dl^{-1}$ for women. Quality of life (QOL) was assessed weekly by using an analogue scale to judge energy, activities of daily living and overall quality of life. Forty-eight patients were entered in the study, 24 in the treatment arm and 24 in the control arm. The prerandomization demographic characteristics and mean laboratory values were comparable in both arms. The mean haemoglobin at completion was 13.6 g $dl^{-1}$ for r-HuEPO-treated patients compared with 11.0 g $dl^{-1}$ for control subjects ($P = 0.0012$). Patients who received r-HuEPO demonstrated a mean weekly haemoglobin increase of 0.41 g $dl^{-1}$ compared with a decrease in mean haemoglobin level in controls for 6 of the 7 weeks of the study (mean weekly decrease of 0.073 g $dl^{-1}$). Target levels of haemoglobin were achieved by 41.6% of r-HuEPO-treated patients compared with none of the control subjects. The mean platelet count declined in both arms of the study with RT but the decline from pretreatment was less rapid in r-HuEPO-treated patients (11.2% decrease) compared with controls (26.3% decrease) and was statistically significant during weeks 4–6. Toxicity was minor with only mild irritation at the injection site. Mean quality of life end points were superior in the treatment arm but not statistically significant. r-HuEPO had a beneficial effect on weekly haemoglobin levels in patients undergoing RT with response rates similar to other studies. There was also a less rapid decline in weekly platelet counts in r-HuEPO-treated patients compared with control subjects. Further studies are needed to address the optimum dose and scheduling as well as the impact of r-HuEPO on clinical outcomes.

**Keywords:** erythropoietin; radiotherapy; anaemia in cancer

Anaemia is a common occurrence in cancer patients at some point in their disease course (Samuels and Bierman, 1956; Hirst, 1986; Abels, 1992*a*; Leitgeh et al, 1994). The presence of anaemia reflects a reduction in red cell mass and a corresponding decrease in the oxygen-carrying capacity of the blood (Bunn, 1991). Although the severity is variable from patient to patient, anaemia can lead to symptoms of fatigue, dyspnoea and loss of appetite and can cause or aggravate cardiovascular and respiratory problems (Ludwig et al, 1993; Leitgeh et al, 1994). The causes of anaemia in cancer patients are often multifactorial including haemorrhage, haemolysis and iron deficiency as well as tumour infiltration of bone marrow and the toxicities of cancer therapy (radiation and chemotherapy) (Abels, 1992*a,b*; Spivak, 1992; Longo, 1993). One component may be related to the so-called 'anaemia of chronic disease' (Lee, 1983; Abels, 1992*b*; Longo, 1993). The anaemia of chronic disease results from decreased erythrocyte lifespan (about 80 days rather than the normal 120 days), impaired flow of iron from macrophages to plasma and an inadequate erythropoetin response to anaemia (Miller et al, 1990; Abels, 1992*a,b*; Kushner, 1992; Dšhrsen and Hossfeld, 1994). In cancer patients erythropoietin production has been found to be particularly depressed compared with patients with either iron deficiency anaemia or other normochromic, normocytic anaemias associated with chronic disease (Miller et al, 1990; Kettelhack et al, 1994). This may be partly related to the inhibitory effect of increased cytokine production of tumour necrosis factor alpha and interleukin 1 on erythropoesis (Dšhrsen and Hossfeld, 1994; Spivak, 1994). Thus, the anaemia of cancer is at least partially due to a deficiency of erythropoietin (Miller et al, 1990; Abels, 1992*a,b*, 1993; Spivak, 1992, 1994).

Erythropoietin is the primary regulatory factor in erythropoiesis (Zanjani and Ascensao, 1989). It is an acidic glycoprotein with an estimated molecular weight of 34 000 that is produced primarily in the kidney (Jacobson et al, 1957; Wang et al, 1985; Spivak, 1989; Zanjani and Ascensao, 1986). Tissue hypoxia induces erythropoietin production which stimulates erythroid progenitor cells (Goldberg et al, 1988; Zanjani and Ascensao, 1989; Spivak, 1992, 1994). Recently, through the use of recombinant technology exogenous erythropoietin has become available. Thus, it is now possible

*Received 9 May 1997*
*Revised 2 November 1997*
*Accepted 3 December 1997*

*Correspondence to:* PJ Sweeney, University of Chicago, Department of Radiation and Cellular Oncology, 5841 S. Maryland Ave, MC 0085, Chicago, IL 60637, USA

**Table 1** Demographic characteristics and prerandomization laboratory values[a]

| Demographic characteristics and laboratory data[b] | Control arm | Treatment arm | P-value |
|---|---|---|---|
| Age (years) | 62.7 | 62.3 | 0.89 |
| Sex | | | |
| Male | 13 | 15 | 0.558 |
| Female | 11 | 9 | |
| Diagnosis | | | |
| Breast cancer | 5 | 5 | 0.532 |
| Lung cancer | 11 | 7 | |
| Prostate cancer | 6 | 10 | |
| Cervix cancer | 1 | 2 | |
| Unknown | 1 | | |
| Previous transfusion | | | |
| Yes | 3 | 2 | 0.64 |
| No | 21 | 22 | |
| Haemoglobin (gm $dl^{-1}$) (men 14–18; women 12–16) | 10.72 | 12.07 | 0.22 |
| RBC count ($\times 10^6$ $mm^{-3}$) (men 4.6–6.2; women 4.2–5.4) | 3.7 | 3.88 | 0.28 |
| WBC count ($\times 10^3$ $mm^{-3}$) (4.8–10.8) | 7.47 | 6.88 | 0.11 |
| Platelets ($\times 10^3$ $mm^{-3}$) (159–400) | 350.7 | 295.38 | 0.11 |
| Reticulocyte count (%) (0.5–1.5) | 1.29 | 1.31 | 0.94 |
| Serum iron (μg $dl^{-1}$) (men 80–160; women 60–135) | 82.58 | 54.95 | 0.43 |
| Serum ferritin (ng $ml^{-1}$) (men 36–262; women 10–155) | 347.76 | 223.85 | 0.11 |
| Total iron-binding capacity (TIBC) (μg $dl^{-1}$) (250–350) | 251.39 | 271.05 | 0.33 |
| Serum folate level (ng $ml^{-1}$) (2.5–17.5) | 25.04 | 17.76 | 0.7 |
| Serum B12 level (pg $ml^{-1}$) (250–1000) | 667.2 | 633.1 | 0.77 |
| Serum erythropoetin level (IU/l) (4–26) | 27.52 | 25.96 | 0.91 |

[a]Units of measurement in parentheses. Normal values in parentheses. [b]Mean laboratory values. Data not available on all patients.

**Figure 1** (**A**) Haemoglobin; (**B**) platelets; (**C**) White blood cell count to manipulate erythropoiesis independently of endogenous erythropoietin production (Spivak, 1994). Recombinant erythropoietin (r-HuEPO) has previously been shown to increase the haematocrit and reduce the transfusion requirement in patients with end-stage renal disease undergoing haemodialysis (Esbach et al, 1989) and in AIDS patients treated with zidovudine (Fischl et al, 1990). In addition, there are now a number of reports on the use of r-HuEPO to correct anaemia in cancer patients (Platanias et al, 1991; Abels, 1992*a,b*, 1993; Miller et al, 1992; Case et al, 1993; Lavey and Dempsey, 1993; Ludwig et al, 1993, 1994; Vijayakumar et al, 1993; Dusenbery et al, 1994; Leitgeh et al, 1994; deCampos et al, 1995). In the largest randomized trial published to date, Abels (1993) demonstrated that r-HuEPO corrected anaemia and reduced transfusion requirements compared with a control group for patients with a variety of cancers undergoing chemotherapy. However, this particular study excluded patients receiving radiotherapy (RT). In view of the above, a prospective, randomized, open-labelled clinical trial was conducted to determine the safety and efficacy of subcutaneous r-HuEPO in cancer patients undergoing RT. A randomized design was chosen to compare weekly changes in blood cell parameters between the treatment and the control group in a prospective and unbiased manner and to separate the toxicities associated with RT from any unexpected toxicities of r-HuEPO administration in the treatment arm. In addition, we sought to assess quality of life measures between those patients in the treatment and control arms. We have previously reported the interim analysis of the first 26 patients entered (Vijayakumar et al, 1993) and now present the final report of the 48 patients who completed this study.

### METHODS AND MATERIALS

The study was initiated in September 1991 and completed in February 1996. The participating institutions were the departments of radiation oncology at the University of Chicago (UC), the

© Cancer Research Campaign 1998

University of California in San Francisco (UCSF) and the Joint Center for Radiation Therapy (JCRT) in Boston. Informed consent was obtained from all patients before randomization. Entry was restricted to patients with either carcinoma of the lung, uterine cervix, prostate or breast who presented for radiation therapy with anaemia. Patients were required to have a Karnofsky performance status of ≥ 70 and a life expectancy of at least 3 months. Only patients who would undergo at least 4 weeks of RT were eligible. Although not specifically considered an exclusion criteria, only three patients in the r-HuEPO arm and two of the control patients received concomitant chemotherapy. Thus, impact of r-HuEPO on patients receiving RT cannot be considered confounded by the addition of concomitant chemotherapy. Anaemia was considered to be haemoglobin ≤ 13 gm $dl^{-1}$ in males and ≤ 12 gm $dl^{-1}$ in female patients. So as to best approximate the anaemia of chronic disease, patients with evidence of iron, folate or $B_{12}$ deficiency or guiaic-positive stool were excluded. Patients were required to have serum iron of > 20%; ferritin > 20 ng $ml^{-1}$; TIBC < 400 µg $dl^{-1}$ and be direct Coomb's test negative. Metastatic disease was not an exclusion criteria except for patients with lung primaries or cerebral metastases.

Patients in the treatment arm received 200 units(U) $kg^{-1}$ of r-HuEPO (Procrit, Ortho Biotech, Raritan, NJ, USA) subcutaneously, five times a week, until the haemoglobin reached its target level. Injections for up to 7 weeks were allowed considering this to be the maximum length of a radical course of RT for the malignancies in this study. The target levels of haemoglobin were 15 gm $dl^{-1}$ for men and 14 gm $dl^{-1}$ for women. Once the target haemoglobin level was achieved, the dose of r-HuEPO was reduced by 50% for maintenance of this level until completion of RT. All patients in the treatment arm received iron (ferrous sulphate, 325 mg. p.o. t.i.d.) supplements during the period of r-HuEPO administration. Iron supplements were used to ensure adequate iron stores during erythropoiesis in order to prevent the development of iron deficiency anaemia (Van Wyck, 1989). Patients in the control arm did not receive iron supplements. Radiotherapy treatment policies were based on the site and stage of the disease and were at the discretion of the treating physician in terms of total dose, fraction size, treatment portals, etc. Complete blood counts (CBC) were obtained prerandomization for baseline values (week 0) and weekly thereafter during the RT. The following parameters were analysed: haemoglobin, total white blood cell count (WBC) and platelets.

A patient self-assessed, subjective quality of life assessment was also obtained on all patients on a weekly basis. Patients were asked to mark their assessment of quality of life in a visual analogue format. Three aspects of quality of life were addressed: energy level, ability to perform activities of daily living and overall quality of life. A computer program was written for converting the subjective quality of life assessment to numerical objective values. At the completion of RT blood counts were obtained on those patients available for follow-up with a maximum follow up time of 18 months.

Patients were randomized between r-HuEPO and control by creating random numbers seperately by disease site and treatment centre in bins of 10 by a computer. The differences between the control and treatment arm of the various CBC parameters were analysed by subtracting the weekly parameter from the baseline value for each patient. A mean of all patients' incremental/decremental values for each parameter was calculated and the difference between the control and treatment arm was tested. Statistical analyses were carried out with the two-tailed *t*-test for two variables and the chi-square test for multiple variables.

**Table 2** Mean changes in weekly haemoglobin levels for the treatment and control arms[a]

| Week studied | Control | r-HuEPO | *P*-value |
|---|---|---|---|
| Baseline | 10.72 | 12.07 | 0.220 |
| Week 1 | 10.37 (16) | 12.51 (16) | 0.030 |
| Week 2 | 10.39 (16) | 13.02 (20) | 0.001 |
| Week 3 | 10.55 (19) | 13.4 (22) | 0.0004 |
| Week 4 | 10.79 (16) | 13.85 (23) | 0.001 |
| Week 5 | 10.67 (15) | 14.38 (21) | 0.000007 |
| Week 6 | 10.63 (17) | 14.38 (19) | 0.0004 |
| Week 7 | 10.52 (15) | 14.69 (13) | 0.00002 |

**Table 3** Weekly mean change in haemoglobin level (g $dl^{-1}$) subtracted from the previous week's mean level[a]

| Week studied | Control | r-HuEPO | *P*-value |
|---|---|---|---|
| Week 1 | −0.347 (16) | 0.44 (16) | 0.03 |
| Week 2 | −0.07 (15) | 0.679 (16) | 0.003 |
| Week 3 | −0.069 (16) | 0.361 (19) | 0.18 |
| Week 4 | 0.309 (16) | 0.525 (22) | 0.32 |
| Week 5 | −0.169 (13) | 0.433 (21) | 0.015 |
| Week 6 | −0.162 (13) | 0.21 (18) | 0.17 |
| Week 7 | −0.029 (14) | 0.1 (12) | 0.73 |

[a]Number in parentheses is sample size for that week.

**Table 4** Mean changes in weekly platelet levels for the r-HuEPO and control arms[a]

| Week studied | Control | r-HuEPO | *P*-value |
|---|---|---|---|
| baseline platelet level | 351 | 295 | 0.11 |
| Week 1 change | 27.2 (15) | −33.0 (16) | 0.17 |
| Week 2 change | −2.1 (15) | −23.4 (18) | 0.57 |
| Week 3 change | −52.6 (18) | −1.2 (20) | 0.06 |
| Week 4 change | −82.2 (14) | −7.9 (22) | 0.03 |
| Week 5 change | −88.3 (14) | −9.8 (18) | 0.004 |
| Week 6 change | −106.8 (16) | −49.3 (18) | 0.05 |
| Week 7 change | −102.1 (15) | −68.6 (13) | 0.190 |

[a]Number in parentheses is sample size for that week.

### RESULTS

A total of 48 patients were entered in the study. Half of the patients were men and half women. There were 37 patients from UC, ten patients from UCSF and one patient from the JCRT. The breakdown by cancer is as follows: lung cancer, 18 patients; breast cancer, ten patients; prostate cancer, 16 patients; and uterine cervix cancer, three patients. There was one patient whose primary diagnosis was unknown. There were six patients who were treated palliatively, the remainder were treated with curative intent. Two patients refused r-HuEPO injections after randomization; one refused after 3 days of injections and the second before any drug had been administered. These patients are excluded from the analysis.

The demographic characteristics and prerandomization laboratory results are shown in Table 1. The only notable laboratory value is the baseline mean haemoglobin level, which is greater by more than 1 g in the treatment arm (12.0 g $dl^{-1}$) than in the control arm (10.7 g $dl^{-1}$). However, this difference is not statistically

© Cancer Research Campaign 1998

**Table 5** Summary of the pre- and post-RT parameters for the r-HuEPO and control arms

| | Control | r-HuEPO | *P*-value |
|---|---|---|---|
| Haemoglobin | | | |
| Pre-RT | 10.72 | 12.07 | 0.22 |
| Post-RT | 11.01 | 13.62 | 0.0012 |
| Platelets | | | |
| Pre-RT | 351 | 295 | 0.11 |
| Post-RT | 258 | 262 | 0.92 |
| WBC | | | |
| Pre-RT | 7.47 | 6.88 | 0.64 |
| Post-RT | 4.32 | 4.3 | 0.98 |

**Table 6** Weekly mean quality of life scores for control and treatment arm

| Week studied | Control | r-HuEPO | *P*-value |
|---|---|---|---|
| Week 1 | 50.0 (15) | 53.6 (17) | 0.74 |
| Week 2 | 45.9 (15) | 52.1 (17) | 0.56 |
| Week 3 | 53.6 (15) | 57.6 (17) | 0.73 |
| Week 4 | 56.3 (15) | 58.5 (17) | 0.84 |
| Week 5 | 56.9 (15) | 64.6 (17) | 0.47 |
| Week 6 | 57.9 (15) | 63.3 (17) | 0.62 |
| Week 7 | 56.3 (14) | 72.7 (13) | 0.15 |

Number in parentheses is sample size for that week.

significant ($P = 0.22$). In general, the two arms are well balanced without significant differences in baseline characteristics. The mean values represented by this table demonstrate a fairly classic picture of the anaemia of chronic disease, specifically, the mean iron and total iron binding capacity (TIBC) values are low–normal and the reticulocyte count and ferritin levels are in the normal range. The mean serum erythropoietin level for the entire group was 26.7 IU ml$^{-1}$. The normal range in adults without anaemia is 4–26 IU per l (Mendenhall et al, 1984; Egrice et al, 1987). Thus, the endogenous erythropoietin level similarly represents the classic picture of the anaemia of malignancy with a value that is elevated but below what would be expected for iron deficiency anaemia (Mendenhall et al, 1984).

Figure 1A shows the treatment and control arms for the weekly haemoglobin levels. The mean haemoglobin in the patients who received r-HuEPO demonstrated a gradual increase from the baseline level during the weeks of the study as shown by the upward slope. The haemoglobin levels of patients in the control arm demonstrated no significant change in mean haemoglobin during this time period and have a static curve. The mean haemoglobin at completion was 13.6 g dl$^{-1}$ for the treated patients compared with a week 0 level of 12.0 g dl$^{-1}$. For the control patients, the completion mean haemoglobin level was 11.0 g dl$^{-1}$ compared with a baseline of 10.7 g dl$^{-1}$. The differences between the r-HuEPO group and control mean haemoglobin levels were statistically significant for each week of the study. These data are also presented in Table 2.

These results might be considered misleading because the higher baseline haemoglobin of the treated patients compared with the control patients (12.0 g dl$^{-1}$ vs 10.7 g dl$^{-1}$) inflates the difference in the mean haemoglobin levels, especially during the later weeks of the study. However, this baseline difference is eliminated when the week-to-week changes in haemoglobin are analysed. This is demonstrated in Table 3 where the weekly mean haemoglobin

**Figure 2** Haemoglobin follow-ups level of the treatment and control patients is subtracted from the previous week's level during each of the 7 weeks of the study and the differences between the two groups tested. For example, the haemoglobin level obtained in week 1 is subtracted from the baseline (week 0) haemoglobin, the haemoglobin from week 2 is subtracted from week 1, etc. This table shows that for each of the 7 weeks of the study there is an increase in haemoglobin from the previous week for those patients who were treated with r-HuEPO. The average net increase in haemoglobin per week is 0.41 g dl$^{-1}$ and is greatest between weeks 0 and 5 before levelling off. The maximum increase for 1 week is between weeks 1 and 2, when the mean haemoglobin increases 0.68 g dl$^{-1}$. Patients in the control arm show a decrease in haemoglobin from the previous week's reading for 6 of the 7 weeks of the study with a range of between 0.03 and 0.35 g dl$^{-1}$.

The target level of haemoglobin of 15 g dl$^{-1}$ for men and 14 g dl$^{-1}$ for women was achieved by 41.6% (10 out of 24) of the patients in the r-HuEPO arm. This includes six men and four women. The mean time to normalization of haemoglobin was 4.2 weeks for men and 3.9 weeks for women. None of the patients in the control group reached these target haemoglobin levels. If the target level of haemoglobin was considered to be 14 gm dl$^{-1}$ for men and 13 g dl$^{-1}$ for women, as is the case in some laboratories, then 65.2% of the r-HuEPO patients achieved this haemoglobin level compared with only one control patient (5.2%). The type of cancer did not have a predictive value on either the pre-RT haemoglobin level or progression of haemoglobin during the RT for either the r-HuEPO or the control group (data not shown).

Follow-up hemoglobin levels were obtained on a subset of patients. The number of patients continually decreased as the interval from the completion of RT increased because of patients dying or being lost to follow-up. As demonstrated in Figure 2, with a maximum interval of 18 months, the mean level of haemoglobin in the r-HuEPO patients is consistently higher than in the control patients. However, the small number of patients in which follow-up haemoglobin levels were available preclude this difference from showing statistical significance.

Figure 1B and C show the changes in platelets and WBC respectively. The mean platelet count in the control patients demonstrated a progressive decline with RT from a week 0 level of 350 to an end of treatment level of 258, a 26.3% decrease. The

© Cancer Research Campaign 1998

mean platelet count in the r-HuEPO patients was 295 at the commencement of RT and 262 at completion, only an 11.2% decrease. A $t$-test for the differences between the pre- and post-RT platelet values for the r-HuEPO and control groups indicates a significantly different rate of decline ($P = 0.03$). This 'positive' effect of r-HuEPO on platelets is shown in Figure 1B as an initial minimal increase in mean platelet count and then a levelling off during weeks 3–5 with a more gradual decline during the final 2 weeks of RT. Table 4 demonstrates that the weekly changes in mean platelet count for the r-HuEPO patients achieve borderline significance during week 3 ($P = 0.060$) and are statistically significant during weeks 4–6 when compared with controls. Figure 1C demonstrates the lack of effect of r-HuEPO on the WBC values. White blood cell counts decreased in both arms of the study with RT. A comparison of the pre- and post-RT parameters for the treatment and control arm are summarized in Table 5.

### Adverse effects/quality of life

One patient in the treatment arm developed pruritis, which cleared within 2 days, and he subsequently continued r-HuEPO administration without further difficulties. Five other patients experienced adverse effects during the trial that were considered unrelated to r-HuEPO. Three patients developed herpes zoster in dermatomes within the radiotherapy field (two were in the control arm and one in the treatment arm). Two patients complained of dyspepsia, attributed to iron supplements, although none discontinued taking iron tablets. No increased skin or other side-effects were observed during RT in the treatment arm.

A quality of life (QOL) survey was completed weekly during RT by all patients. Three aspects of quality of life were addressed: energy level, ability to perform activities of daily living and overall quality of life. Our results demonstrate that the answers for all patients were highly correlated for the three aspects meaning an individual patient generally recorded a similar score for each of the three questions. Thus, the results are presented as a single QOL parameter. Table 6 shows the weekly mean QOL scores for the treatment and control groups. Note that for each week of the study there is a more favourable numerical assessment of QOL in the r-HuEPO arm compared with the control arm. These weekly differences did not achieve statistical significance, however, because there was a large variation in individual QOL scores in the control arm. Nevertheless, as the table demonstrates, there is a strong trend towards better QOL in patients who received r-HuEPO.

### DISCUSSION

For cancer patients receiving RT, anaemia has been correlated with unfavourable outcomes when compared with non-anaemic patients for a number of disease sites (Dische, 1991). Most of the literature concerns malignancies of the uterine cervix (Evens and Bergso, 1965; Hierlihy et al, 1969; Vigerio et al, 1973; Bush et al, 1978; Dische et al, 1983; Mendenhall et al, 1984; Bush, 1986; Girinski et al, 1989; Rader, 1990) and head and neck (Blitzer et al, 1984; Overgard et al, 1989; Dubray et al, 1996). How anaemia is causally related to poor outcome is not entirely clear but it is probably both a marker of advanced tumour and indicative of some degree of tissue hypoxia. Because it is an axiom in radiobiology that hypoxic cells are less radioresponsive than aerated cells (Gray et al, 1953; Palcic and Skarsgard, 1984), it has been standard treatment to transfuse anaemic cancer patients to some arbitrary haemoglobin level in an effort to improve tissue oxygenation as well as to enhance patient comfort (Poskitt, 1987). Despite this practice, the routine use of blood transfusion to improve serum haemoglobin level has risks, including viral infection (HIV and hepatitis) and transfusion reaction (Bove, 1987; Poskitt, 1987; Levine and Vijayakumar, 1993). In addition, there is the potential down-regulation of host cellular immune function with transfusion (Blumberg and Heal, 1990). A number of retrospective reviews have documented adverse outcomes (inferior overall and disease-free survival) in patients receiving blood transfusions for a variety of malignancies including colon/rectum, lung, prostate, uterine cervix, breast and soft tissues (Rosenberg et al, 1985; Tartter et al, 1985; Arnoux et al, 1988; Blumberg et al, 1988; Corman et al, 1988; Heal et al, 1988; Moores et al, 1989; Wobbes et al, 1989; Little et al, 1990; McClinton et al, 1990; Casper et al, 1991). Thus, although transfusion can improve tissue oxygenation and potentially enhance the effectiveness of RT, any radiobiological gain might be mitigated by the adverse effects of transfusion on tumour immunosurveillance (Levine and Vijayakumar, 1993). Recombinant erythropoetin offers the possibility of correcting cancer-related anaemia without subjecting patients to the risks of transfusion.

The results of this study demonstrate the use of r-HuEPO in improving the haemoglobin levels in anaemic cancer patients undergoing RT with an average increase of about 0.4 g $dl^{-1}$ per week during treatment. This improvement in haemoglobin was seen in every week of the study and was greatest during the second week with a mean increase of 0.68 g $dl^{-1}$. Although the mean haemoglobin level at completion was 13.6 g $dl^{-1}$, which is below the targeted levels of 15 g $dl^{-1}$ for men and 14 g $dl^{-1}$ for women, this represents an improvement over patients in the control arm who showed no change with RT. Furthermore, when the target levels are reduced to the more realistic values of 14 g $dl^{-1}$ for men and 13 g $dl^{-1}$ for women, 65% of the r-HuEPO patients achieved these values compared with only 5.2% of control patients.

These findings in our randomized study confirm the erythropoietic effect of r-HuEPO demonstrated in previous non-randomized studies of anaemic cancer patients undergoing RT as well as our preliminary report (Lavey and Dempsey, 1993; Vijayakumar et al, 1993; Dusenbery et al, 1994). Lavey and Dempsey (1993) described an improvement in mean haemoglobin level from 11.9 g $dl^{-1}$ to 15.1 g $dl^{-1}$ in 20 patients who received r-HuEPO while undergoing RT for supradiaphragmatic tumours (Kushner, 1992). This is an approximate haemoglobin increase of 0.45 g $dl^{-1}$ per week over a 7-week RT treatment course. Similarly, Dusenbery et al (1994) showed an improvement in mean haemoglobin from 10.3 to 13.2 g $dl^{-1}$ in 15 patients with uterine cervix cancer who received r-HuEPO during RT, which is an increase of 0.5 g $dl^{-1}$ per week (Dusenbery et al, 1994). These studies demonstrate an increase in mean haemoglobin that is slightly greater than the average weekly haemoglobin increase in our study of 0.41 g $dl^{-1}$. This may be explained by the fact that patients in each of these studies received r-HuEPO as early as 10 days before the start of RT, whereas patients in our study started r-HuEPO during the first week of RT.

Despite the favourable effect on mean haemoglobin levels seen in these studies the optimum dose and scheduling of r-HuEPO for cancer patients has not been established. Our study used 200 units $kg^{-1}$ for 5 consecutive days as the initial dose and then decreased the dose by 50% once the anaemia was corrected. This was similar to the dose and scheduling of Dusenbery et al (1994) and reflects the favourable results of patients with chemotherapy-induced

© Cancer Research Campaign 1998

anaemia reported by Platanias et al (1991) in a dose escalation study of r-HuEPO that demonstrated the highest response rates in patients who received either 200 or 300 U $kg^{-1}$ for 5 days rather than lower dose levels. However, Lavey and Dempsey (1993) used 300 U $kg^{-1}$ three times only during the first week and then 150 U $kg^{-1}$ (three times a week) for the remainder of the RT. This dosing schedule is fairly close to that used in the two largest experiences of r-HuEPO in patients with cancer-related anaemia (Abels, 1992*a,b*, 1993; Ludwig et al, 1993, 1994) including patients receiving combination chemotherapy (Case et al, 1993). Response rates in these studies are defined differently but are approximately in the 40 to 50% range of patients tested, which is similar to our results. Thus, the correct r-HuEPO dose for RT-treated cancer patients is probably at least 150 U $kg^{-1}$ given three times per week and preferably at least 1 week before the commencement of RT. The dose can be reduced once the anaemia is corrected for the remainder of the RT.

There were no adverse effects related to the r-HuEPO in our patients, except for minor irritation at the injection site. This was similar to the experience of Lavey and Dempsey (1993). Dusenbery et al (1994) did describe deep venous thrombosis (DVT) occurring in 4 of 15 patients either during or just thereafter r-HuEPO administration (Dusenberg et al, 1994). Platanias et al (1991) also reported DVT occurring in two of the eight patients treated at the highest dose level (300 U $kg^{-1}$) of r-HuEPO and Miller et al (1990) had 1 patient out of 21 develop DVT, although this was in the lowest dose group (25 U $kg^{-1}$). In a placebo-controlled trial by Case et al (1993) the only statistically significant difference in the incidence of any adverse effect in r-HuEPO-treated patients compared with controls was diarrhoea and diaphoresis. Other reports have similarly shown the drug to be fairly well tolerated at the dose levels used for cancer patients (Abels, 1993; Ludwig et al, 1993; deCampos et al, 1995).

We compared quality of life end points between r-HuEPO-treated and control patients and found that for each week of study there was a more favourable QOL assessment in the treated patients, although wide variation of responses in the control group precluded statistical significance. Nevertheless, this trend toward improved QOL in r-HuEPO-treated patients is reflected in the literature with most (Abels, 1992*b*, 1993; Case et al, 1993; Ludwig et al, 1993; Leitgeh et al, 1994) but not all (Dusenberg et al, 1994) series showing a benefit in various quality of life parameters over concurrent or historical controls.

One unexpected finding in our study was the positive effect of r-HuEPO on platelets. The gradual decline in platelets with partial body irradiation has been previously reported (Yang et al, 1995). Although the r-HuEPO-treated patients in this study demonstrated a decline in mean platelet level with RT there was clearly seen a less rapid decline than in the control group. This effect has also been described by de Campos et al (1995), who noted fewer platelet transfusions in patients with small-cell-lung cancer treated chemotherapy and RT who received r-HuEPO compared with patients who did not. This thrombopoietic effect of r-HuEPO and its therapeutic implications has been discussed in detail by one of us (SV) in a separate communication (Vijayakumar et al, 1998).

In summary, this study confirms the findings of our preliminary report of the beneficial effect of r-HuEPO on haemoglobin levels in patients undergoing RT for a variety of malignancies. The mean haemoglobin increase in those patients who received r-HuEPO was 0.41 g $dl^{-1}$ per week. By our stringent definition of response (target haemoglobin level of 15 g $dl^{-1}$ for men and 14 g $dl^{-1}$ for women), 41.6% of patients responded, which is equivalent to other studies of anaemic cancer patients. Further, r-HuEPO was found to be safe at the dose levels used and had a favourable impact on quality of life end points. Future studies need to address the optimum dose and scheduling for RT patients and to prospectively compare outcomes from treatment of patients receiving r-HuEPO with control patients.

### ACKNOWLEDGEMENT

This study was supported by a grant from Ortho Biotech, Raritan, New Jersey, USA.

### REFERENCES

Abels RI (1992*a*) Recombinant human erythropoetin in the treatment of cancer. *Acta Haematologica* **87** (suppl. 1): 4–11

Abels RI (1992*b*) Use of recombinant human erythropoetin in the treatment of anemia in patients who have cancer. *Sem Oncol* **19** (suppl. 3): 29–35

Abels RI (1993) Erythropoetin for anemia in cancer patients. *Eur J Cancer* **29A** (suppl. 2): 2–8

Arnoux R, Corman J, Peloquin A, Smeesters C and St-Louis G (1988) Adverse effect of blood transfusions on patient survival after resection of rectal cancer. *Can J Surg* **31**: 121–126

Blitzer PH, Wang CC and Suit HD (1984) Blood pressure and hemoglobin concentration: Multivariate analysis of local control after irradiation for head and neck cancer. *Int J Radiat Oncol Biol Phys* **10** (suppl. 2): 98

Blumberg N and Heal JM (1990) Transfusion-induced immunomodulation and its possible role in cancer recurrence and perioperative bacterial infection. *Yale J Biol Med* **63**: 429–433

Blumberg N, Agarwal MM and Chuang C (1988) A possible association between survival time and transfusion in cervical cancer. *Yale J Biol Med* **61**: 493–500

Bove JR (1987) Transfusion-associated hepatitis and AIDS. *N Engl J Med* **317**: 292–295

Bush RS (1986) The significance of anemia in clinical radiotherapy. *Int J Radiat Oncol Biol Phys* **12**: 2047–2050

Bush RS, Jenkin RDT, Allt WEC, Beale FA, Bean H, Dembo AJ and Pringle JF (1978) Definitive evidence for hypoxic cells influencing cure in cancer therapy. *Br J Cancer* **37** (suppl. III): 302–306

Bunn HF (1991) Anemia. In *Harrison's Principles of Internal Medicine*, Wilson JD, Braunwald E, Isselbacher KJ, Petersdorf RG, Martin JB, Fauci AS and Boot RK (eds). McGraw-Hill: New York, pp. 344–348

Case DC, Bukowski RM, Carey RW, Fishkin EH, Henry DH, Jacobson RJ, Jones SE, Keller AM, Kugler JW, Nichols CR, Salmon SE, Silver RT, Storniolo AM, Wampler GL, Dooley CM, Larholt KM and Abels RI (1993) Recombinant human erythropoetin therapy for anemic cancer patients on combination chemotherapy. *J Natl Cancer Inst* **85**: 801–806

Casper ES, Gaynor JJ, Hadju SI, Magill GB, Tan C, Friedrich C and Brennan MF (1991) A prospective randomized trial of adjuvant chemotherapy with bolus versus continuous infusion of doxorubicin in patients with high grade extremity soft tissue sarcoma and an analysis of prognostic factors. *Cancer* **68**: 1221–1229

Corman J, Arnoux R, Peloquin A, St-Louis G and Smeesters C (1988) Perioperative blood transfusions and colorectal cancer outcome. *Transplant Proc* **20**: 1128–1129

deCampos E, Radford J, Steward W, Milroy R, Dougal M, Swindell R, Testa N and Thatcher N (1995) Clinical and in-vitro effects of recombinant human erythropoietin in patients receiving intensive chemotherapy for small-cell lung cancer. *J Clin Oncol* **13**: 1623–1631

Dische S (1991) Radiotherapy and anemia – the clinical experience. *Radiother Oncol* **20**: 35–40

Dische S, Anderson PJ, Sealy R and Watson ER (1983) Carcinoma of the cervix – anemia, radiotherapy and hyperbaric oxygen. *Br J Radiol* **56**: 251–255

Dšhrsen U and Hossfeld DK (1994) Hematopoietic growth factors and the treatment of tumor-associated anemias. *Ann Hematol* **69**: 213–221

Dubray B, Mosseri V, Brunin F, Jaulerry C, Poncet P, Rodriguez J, Brugšre J, Point D, Giraud P and Cosset J (1996) Anemia is associated with lower local–regional control and survival after radiation therapy for head and neck cancer: a prospective study. *Radiology* **201**: 553–558

Dusenbery KE, McGuire WA, Holt PJ, Carson LF, Fowler JM, Twiggs LB and Potish RA (1994) Erythropoietin increases hemoglobin during radiation therapy for cervical cancer. *Int J Radiat Oncol Biol Phys* **29**: 1079–1084

Egrie JC, Cotes PM, Lane J, Das Gaines RE and Tam RC (1987) Development of radioimmunoassays for human erythropoietin using recombinant erythropoietin as tracer and immunogen. *J Immunol Methods* **99**: 235–241

Esbach JW, Abduljadi MH, Browne JK, Delano BG, Downing MR, Egrie JC, Evans RW, Friedman EA, Graber SE, Haley NR, Korbett S, Krantz SB, Lundin AP, Nissenson AR, Ogden DA, Paganini EP, Rader B, Rutsky EA, Stivelman J, Stone WJ, Teschan P, Van Stone JC, Van Wyck DB, Zuckerman K and Adamson JW (1989) Recombinant human erythropoietin in anemic patients with end-stage renal disease. Results of a phase III multicenter clinical trial. *Ann Int Med* **111**: 992–1000

Evans J and Bergso P (1965) The influence of anemia on the results of radiotherapy in carcinoma of the cervix. *Radiology* **48**: 709–717

Fischl M, Galpin JE, Levine JD, Groopman JE, Henry DH, Kennedy P, Miles S, Robbins W, Starrett B, Zalusky R, Abels RI, Tsai HC and Rudnick S (1990) Recombinant human erythropoietin for patients with AIDS treated with zidovudine. *N Engl J Med* **322**: 1488–1493

Girinski T, Pejovic-Lenfant MH, Bourhis J, Campana F, Cosset JM, Petit C, Malaise EP, Haie C, Gerbaulet A and Chassagne D (1989) Prognostic value of hemoglobin concentrations and blood transfusions in advanced carcinoma of the cervix treated by radiation therapy: results of a prospective study of 386 patients. *Int J Radiat Oncol Biol Phys* **16**: 37–42

Goldberg MA, Dunning SP and Bunn HF (1988) Regulation of the erythropoietin gene: Evidence that the oxygen sensor is a heme protein. *Science* **242**: 1412

Gray LH, Conger AD, Ebert M, Hornsey S and Scott OCA (1953) The concentration of oxygen dissolved in tissues at the time of irradiation as a factor in radiotherapy. *Br J Radiol* **26**: 638–648

Heal JM, Chuang C and Blumberg N (1988) Perioperative blood transfusions and prostate cancer recurrence and survival. *Am J Surg* **156**: 374–379

Hierlihy P, Jenkin RDT and Stryker JA (1969) Anemia as a prognostic factor in cancer of the cervix: a preliminary report. *Can Med Ass J* **100**: 1100–1102

Hirst DG (1986) Anemia: Problem or opportunity in radiotherapy? *Int J Radiat Oncol Biol Phys* **12**: 2009–2017

Jacobson LO, Goldwasser E, Fried W and Plzak L (1957) Role of the kidney in erythropoiesis (letter). *Nature* **179**: 633–635

Kettelhack C, Schöter D, Matthias D and Schlag PM (1994) Serum erythropoietin levels in patients with solid tumors. *Eur J Cancer* **30A**: 1289–1291

Kushner JB (1992) Hypochromic anemias. In *Cecil Textbook of Medicine*, Wyngaarden JB, Smith LH and Bennett JC (eds). WB Saunders: Philadelphia

Lavey RS and Dempsey WH (1993) Erythropoietin increases hemoglobin in cancer patients during radiation therapy. *Int J Radiat Oncol Biol Phys* **27**: 1147–1152

Lee G (1983) The anemia of chronic disease. *Semin Hematol* **20**: 61–80

Leitgeh C, Pecherstorfer M, Fritz E and Ludwig H (1994) Quality of life in chronic anemia of cancer during treatment with recombinant human erythropoietin. *Cancer* **73**: 2535–2542

Levine EA and Vijayakumar S (1993) Blood transfusion in patients receiving radical radiotherapy: a reappraisal. *Onkologie* **16**: 79–87

Little AG, Wu H-S, Ferguson MK, Ho C-H, Bowers VD, Segalin A and Staszek VM (1990) Perioperative blood transfusion adversely affects prognosis of patients with stage I non-small cell lung cancer. *Am J Surg* **160**: 630–633

Longo DL (1993) Anemia in patients with cancer. *Clin Oncol Alert* (suppl.)

Ludwig H, Leitgeb C, Fritz E, Krainer M, Kšhrer I, Kornek G, Sagaster P and Weissman A (1993) Erythropoietin treatment of chronic anemia of cancer. *Eur J Cancer* **29A** (suppl. 2): 8–12

Ludwig H, Fritz E, Leitgeb C, Pecherstorfer M, Samonigg H and Schuster J (1994) Prediction of response to erythropoietin treatment in chronic anemia of cancer. *Blood* **84**: 1056–1063

McClinton S, Moffat LEF, Scott S, Urbaniak SJ and Kerridge DF (1990) Blood transfusion and survival following surgery for prostatic carcinoma. *Br J Surg* **77**: 140–142

Mendenhall WM, Thar TL, Bova FJ, Marcus RB, Morgan LS and Million RR (1984) Prognostic and treatment factors affecting pelvic control of Stage IB and IIA-B carcinoma of the intact uterine cervix treated with radiation therapy alone. *Cancer* **53**: 2649–2654

Miller CB, Jones RJ, Plantadosi S, Abeloff MD and Spivak JL (1990) Decreased erythropoietin response in patients with anemia of cancer. *N Engl J Med* **322**: 1689–1692

Miller CB, Platanias LC, Mills SR, Zahurak ML, Ratain MJ, Ettinger DS and Jones RJ (1992) Phase I–II trial of erythropoietin in the treatment of cisplatin-associated anemia. *J Natl Cancer Inst* **84**: 98–103

Moores DWO, Piantadosi S and McKneally MF (1989) Effect of perioperative blood transfusion on outcome in patients with surgically resected lung cancer. *Ann Thorac Surg* **47**: 346–351

Overgard J, Hansen HS, Anderson AP, Hjelm-Hansen M, Jørgensen K, Sandberg E, Berthelsen A, Hammer R and Pedersen M (1989) Misonidazole combined with split-course radiotherapy in the treatment of invasive carcinoma of larynx and pharynx: Report from the DAHANCA 2 Study. *Int J Radiat Oncol Biol Phys* **16**: 1065–1068

Palcic B and Skarsgard LD (1984) Reduced oxygen enhancement ratio at low doses of ionizing radiation. *Radiat Res* **100**: 328–339

Platanias LC, Miller CB, Mick RM, Hart RD, Ozer H, McEvilly JM, Jones RJ and Ratain MJ (1991) Treatment of chemotherapy-induced anemia with recombinant human erythropoietin in cancer patients. *J Clin Oncol* **9**: 2021–2026

Poskitt TR (1987) Radiation therapy and the role of red blood cell transfusion. *Cancer Invest* **5**: 231–236

Rader JS, Haraf DJ, Halpern HJ, Rotmensch J, Spelbring DR, Sutton H, Javaheri G and Weichselbaum RW (1990) Radiation therapy in the treatment of cervical cancer: The University of Chicago/Michael Reese Hospital experience. *J Surg Oncol* **44**: 157–165

Rosenberg SA, Seipp CA, White DE and Wesley R (1985) Perioperative blood transfusions are associated with increased rates of recurrence and decreased survival in patients with high-grade soft-tissue sarcomas of the extremities. *J Clin Oncol* **3**: 693–709

Samuels AJ and Bierman HR (1956) Anemia in patients with neoplastic disease. *Calif Med* **84**: 180–184

Spivak JL (1989) Erythropoietin: a brief review. *Nephron* **52**: 289–294

Spivak J (1992) The application of recombinant erythropoietin in anemic patients with cancer. *Sem Oncol* **19** (suppl. 8): 25–28

Spivak JL (1994) Editorial: Recombinant human erythropoietin and the anemia of cancer. *Blood* **84**: 997–1004

Tartter PI, Burrows L, Papatestas AE, Lesnick G and Aufses AH Jr (1985) Perioperative blood transfusion has prognostic significance for breast cancer. *Surgery* **97**: 225–229

Van Wyck DB (1989) Iron management during recombinant human erythropoietin therapy. *Am J Kidney Dis* **14** (suppl. 1): 9–13

Vigario G, Kurohara SS and George FW (1973) Association of hemoglobin levels before and during radiotherapy with prognosis in uterine cervix cancer. *Radiology* **106**: 649–652

Vijayakumar S, Roach M, Wara W, Chan SK, Ewing C, Rubin S, Sutton H, Halpern H, Awan A, Houghton A, Quiet C and Weichselbaum R (1993) Effect of subcutaneous recombinant human erythropoietin in cancer patients receiving radiotherapy: Preliminary results of a randomized, open-labeled, phase II trial. *Int J Radiat Oncol Biol Phys* **26**: 721–729

Vijayakumar S, Nautiyal J, Roach M, Marcus KC, Vaida F and Wara W (1998) Beneficial effects of high doses of human recombinant erythropoietin on platelets: An unexpected finding of a randomized, open labeled clinical study in cancer patients. *Radiat Oncol Invest*

Wang FF, Kung CK and Goldwasser E (1985) Some chemical properties of human erythropoietin. *Endocrinology* **116**: 2286–2292

Wobbes T, Joosen KHG, Kuypers HHC, Beerthuuizen GIJM and Theeuwes GM (1989) The effect of packed cells and whole blood transfusions on survival after curative resection for colorectal carcinoma. *Dis Colon Rectum* **32**: 743–748

Yang FY, Vaida F, Ignacio L, Houghton A, Nautiyal J, Halpern H, Sutton H and Vijayakumar S (1995) Analysis of weekly complete blood counts in patients receiving standard fractionated partial body radiation therapy. *Int J Radiat Oncol Biol Phys* **33**: 607–617

Zanjani ED and Ascensao JL (1989) Erythropoietin. *Transfusion* **29**: 46–57

© Cancer Research Campaign 1998